# Comparative transcriptomic insights into molecular mechanisms of the susceptibility wheat variety MX169 response to *Puccinia striiformis* f. sp. *tritici* (*Pst*) infection

**DOI:** 10.1128/spectrum.03774-23

**Published:** 2024-06-25

**Authors:** Xuan Lv, Jie Deng, Congying Zhou, Ahsan Abdullah, Ziqian Yang, Zhifang Wang, Lujia Yang, Baoqiang Zhao, Yuchen Li, Zhanhong Ma

**Affiliations:** 1 Department of Plant Pathology, Ministry of Agriculture and Rural Affairs (MOA) Key Lab of Pest Monitoring and Green Management, College of Plant Protection, China Agricultural University, Beijing, China; University of Mississippi, University, Mississippi, USA

**Keywords:** RNA-seq analysis, wheat stripe rust, differentially expressed genes, susceptibility gene

## Abstract

Stripe rust of wheat is caused by the fungal pathogen *Puccinia striiformis* f. sp. *tritici* (*Pst*). Breeding durably resistant wheat varieties by disrupting the susceptibility (*S*) gene has an important impact on the control of wheat stripe rust. Mingxian169 (MX169) showed strong stripe rust susceptibility to all the races of *Pst*. However, molecular mechanisms and responsive genes underlying susceptibility of the wheat variety MX169 to *Pst* have not been elucidated. Here, we utilized next-generation sequencing technology to analyze transcriptomics data of “MX169” and high-resistance wheat “Zhong4” at 24, 48, and 120 h post-inoculation (hpi) with *Pst*. Comparative transcriptome analysis revealed 3,494, 2,831, and 2,700 differentially expressed genes (DEGs) at different time points. We observed an upregulation of DEGs involved in photosynthesis, flavonoid biosynthesis, pyruvate metabolism, thiamine metabolism, and other biological processes, suggesting their involvement in MX169’s response to *Pst*. DEGs encoding transcription factors were also identified. Our study suggested the potential susceptibility gene resources in MX169 related to stripe rust response could be valuable for understanding the mechanisms involved in stripe rust susceptibility and for improving wheat resistance to *Pst*.

**IMPORTANCE:**

Our study suggests the potential susceptibility gene resources in MX169 related to stripe rust response could be valuable for understanding the mechanisms involved in stripe rust susceptibility and for improving wheat resistance to *Pst*.

## INTRODUCTION

Wheat stripe rust, caused by *Puccinia striiformis* f. sp. *tritici* (*Pst*), is one of the most devastating wheat diseases. Therefore, the current focus of wheat research is on reducing yield loss due to diseases and ensuring wheat yield. Planting disease-resistant wheat varieties has proven to be an economical and effective way to control wheat stripe rust, as demonstrated by numerous wheat production practices both domestically and internationally (1). Due to the rapid variation of pathogen virulent races, the newly generated races can overcome the resistance of the corresponding genes, resulting in an increasingly fierce competition between the host and the pathogen (2). The breeding speed of disease-resistant varieties lags behind the rapid replacement of dominant races, so it is very important to select wheat varieties with more durable resistance.

Up to now, there are two main research ideas for developing wheat varieties that can be used to control the occurrence and development of stripe rust. The first idea is to identify disease resistance (*R*) genes and apply them to breeding efforts. At present, over 80 wheat stripe rust resistance genes (*Yr*) have been officially named internationally, such as seedling resistance genes (*Yr1-Yr10*, *Yr15*, *Yr17*, *Yr73*, *Yr74*), adult plant resistance genes (*Yr11-Yr14*, *Yr46*, *Yr48*, *Yr49 and Yr52*, *Yr54*, *Yr56*, *Yr59*, *Yr62*), and some provisional stripe rust resistance genes (3–5). So far, a small number of genes have been successfully isolated by researchers. Among them, *Yr18* (*Yr18/Lr34/Pm38/Lr34/Sr57/Bdv1/Ltn1*) also has excellent resistance to wheat leaf rust, stem rust, and powdery mildew (6). As previously mentioned, the rapid mutation of virulent races presents a significant challenge to many current cultivars that contain resistance genes (7). Researchers have developed identification host systems through field trials, such as a set of 18 yellow rust (*Yr*) single-gene lines that was established to differentiate races of *Pst* (8). These wheat lines were utilized to analyze the current prevalent races and composition of *Pst*, achieving monitoring of virulent races (9, 10). Over the past decade, researchers across the globe have tested the virulence of collected *Pst* samples on wheat cultivars with different resistance genes (11–13). It was confirmed that with the emergence of numerous new *Pst* physiological races, many existing *Yr* genes (such as *Yr6-Yr8*, *Yr17*, *Yr43*, *Yr44*) have been overcome, exhibiting virulent to wheat varieties. Notably, the *Yr5* and *Yr15* have consistently demonstrated effective resistance across regions, highlighting their significance in developing durable rust-resistant wheat. However, in the latest two studies, researchers from China and Turkey, respectively, discovered new races with virulence toward the *Yr5* (14, 15). Developing wheat varieties with broad-spectrum or durable resistance is crucial in the face of emerging physiological races, which pose a significant threat to wheat production. However, the other research idea is to destroy host susceptibility (*S*) genes to obtain durable disease resistance. In light of the abovementioned facts, exploring and eliminating wheat susceptibility (*S*) genes are new angles for creating durable resistant wheat varieties.

So far, there are limited reports on the identification of the *S* gene (e.g., *Mlo*, *TaPsIPK1* in wheat). Nonetheless, numerous studies have been conducted in this area. Cherif et al. provided a new clue for exploring the candidate susceptibility genes and pathways in the interaction between wheat grain and *Fusarium* by transcriptome analysis (16). Su et al. demonstrated that *TaHRC* encodes a susceptibility nuclear protein to wheat head blight and that the disruption of *TaHRC* increases resistance effectively (17). To date, numerous studies have shown a great potential of discovering potential susceptible genes by comparing the gene expression differences between resistant and susceptible varieties. Luo et al. used expressed sequence tags (ESTs) technology to study wheat gene expression inoculated by *Blumeria graminis*, and screened genes related to resistance by subtractive hybridization (18). Zhang et al. obtained differentially expressed sequences through the analysis of the cDNA library of wheat–stripe rust affinity interaction and identified *TaNAC2* as a negative regulator during early stages of wheat*–Pst* interaction with potential susceptibility gene function (19). *TaSTP6* was found to be an abscisic acid (ABA)-induced sugar transporter, which promotes wheat susceptibility to stripe rust (20).

With its highly sensitive and comprehensive features, RNA sequencing (RNA-seq) has become one of the important means of transcriptome analysis strategy (21). In recent years, this technology has been widely used to study the mechanisms associated with interactions between wheat and pathogens (22–24). For example, Strau et al. used RNA-seq technology to isolate and identify a gene *Bs4C*, which is a *Xanthomonas* TAL-effector-activated resistance gene in a large-crop genome (25). With the help of the transcriptome analysis, researchers investigated the response of different resistant wheat varieties to *Rhizoctonia cerealis* infection, and these findings provide further opportunities for exploring the sheath blight resistance mechanism in wheat (26, 27).

In this study, the main focus was to investigate the compatible interaction between wheat and stripe rust, with a particular interest in identifying the key genes involved in this process. To this end, two representative experimental materials were selected, i.e., MX169 and Zhong4. MX169 is a susceptible winter wheat cultivar having no stripe rust resistance genes according to previous research (27); whereas, Zhong4 (Jian15 [J15]) is a highly resistant variety, is one of the important identification hosts of the physiological race of Chinese wheat stripe rust, and has shown resistance to all prevalent races of wheat stripe rust. Due to the outstanding comprehensive agronomic traits and strong resistance of Zhong4 to various wheat diseases, it has become an important germplasm resource for wheat breeding in China and all over the world (28, 29). These two representative experimental materials (i.e., MX169 and Zhong4) with different affinities, genetic backgrounds, and disease resistance phenotypes provided new ideas to explore the key gene of wheat in the interaction between wheat and stripe rust. Therefore, the current comprehensive study was planned to compare the gene expression for both varieties at different time intervals after the plants were inoculated with *Pst*. From this, a comparative transcriptome analysis of MX169 and Zhong4 leaves under *Pst* treatment was performed to find out the potential genes in the susceptible and resistant variety. The main purpose was to identify the changes in transcript expression patterns and find the response mechanisms of susceptible varieties to *Pst*. Moreover, this study also aimed to identify possible susceptibility genes in the stripe rust response through comparison analysis.

## MATERIAL AND METHODS

### Plant materials and pathogen stress treatment

The winter wheat MX169 shows high susceptibility to all races of *Pst*. The wheat Zhong4 is an excellent cultivar that shows high stripe rust resistance in the field and has also been widely used as a parent in wheat breeding programs. The *Pst* race CYR34 and the wheat germplasm were obtained from the Plant Disease Epidemiology Laboratory, CAU. The 10-day-old nine wheat seedlings of each MX169 and Zhong4 were brush inoculated with CYR34 urediniospores, and three wheat seedlings for each variety were kept as control (non-inoculated). Following *Pst* inoculation, the leaf samples (consisting of three biological replicates) were harvested at 24, 48, and 120 h post-inoculation (hpi). The developmental period of three time points was about from 2.5-leaf to the 3.4-leaf stage (The wheat seedlings had 2.5 to 3.4 leaves on their main stem). All samples were frozen immediately in liquid nitrogen and were stored at −80°C. After monitoring, the MX169 samples were fully sporulating and were then used for the experiment.

### RNA isolation and RNA-seq

RNA was extracted using the TRIzol method (Invitrogen, CA, USA) and were treated with RNase-free DNase I (Takara, Kusatsu, Japan) according to the manufacturer’s instructions. RNA concentration and purity were measured using a NanoDrop spectrophotometer (Thermo Fisher Scientific, DE, USA), and the quality of the RNA was analyzed on an Agilent 2100 Bioanalyzer (Agilent Technologies, CA, USA). In total, 18 constructed libraries were sequenced on the Illumina Novaseq 6000 platform to generate 150 bp paired-end reads. Because of the negative effect of the bioinformatics analysis, raw reads with adaptor sequences, an N ratio higher than 10%, and low-quality sequences were removed using Trimmomatic (v0.33). The database was submitted to the NCBI under accession number PRJNA975982.

### Alignment of RNA-seq reads and identification of differentially expressed genes (DEGs)

The generated clean reads were aligned to the wheat reference genome of “*Triticum aestivum*” (ftp://ftp.ensemblgenomes.org:21/pub/plants/release-47/fasta/triticum_aestivum/) using STAR (v2.5.2b) (30). The highly qualified unique mapped read counts were used in the fragments per kilobase per million mapped reads (FPKM) calculation by HTSeq software (v 0.5.4p3) (31). DESeq (1.10.1) was used to analyze differential gene expression; wheat reference genes with a Q-value ≤ 0.001 were considered as DEGs (32).

### GO terms and KEGG pathways analysis

Gene Ontology (GO) function enrichment of the DEGs was performed using David software (33). This software was also used to test the statistical enrichment of differential expression genes in KEGG pathways (34). The molecular function category exhibited the highest enrichment of GO terms, with the most significant enrichment observed in the 48 h post-inoculation group. GO terms and KEGG pathways with false discovery rate (FDR)-corrected Q-values ≤ 0.05 were considered to be significantly enriched.

### Gene set enrichment analysis (GSEA) and plant transcription factor analysis

We performed GSEA with the software GSEA (v2.2.4). The “MX169” and “Zhong4” samples at 24, 48, and 120 hpi were compared separately. Normalized enrichment score (NES), nominal *P*-value (NOM p-val), and FDR were used to quantify enrichment magnitude and statistical significance, respectively. The gene sets under the pathways with |NES| > 1, NOM p-val < 0.05, and FDR < 0.25 were considered significant. Transcription factor analysis was performed on the shared upregulated differential genes, which were obtained from 24, 48, and 120 hpi. We used Blast to obtain the transcription factors by comparing these genes with the Plant Transcription Factor Database 4.0 (http://planttfdb.cbi.pku.edu.cn/).

### Quantitative real-time PCR (qRT-PCR)

Five DEGs were selected for validation of RNA-seq results by qRT-PCR. Gene-specific primers were designed by Primer Premier 6 (Table S2). qPCR was performed on Quant Studio3 according to the manufacturer’s instructions. PCRs included 1 µL of cDNA, 7.5 µL of 2× Master Mix, and 0.3 µL of each primer in a final volume of 15 µL. The 2^-ΔΔCT^ method was used to normalize and calibrate transcript values relative to the endogenous β-actin control (35). Three biological replicates were used for each time point.

## RESULTS

### Transcriptome analysis of the two wheat varieties upon *Pst* inoculation

**Fig 1:**
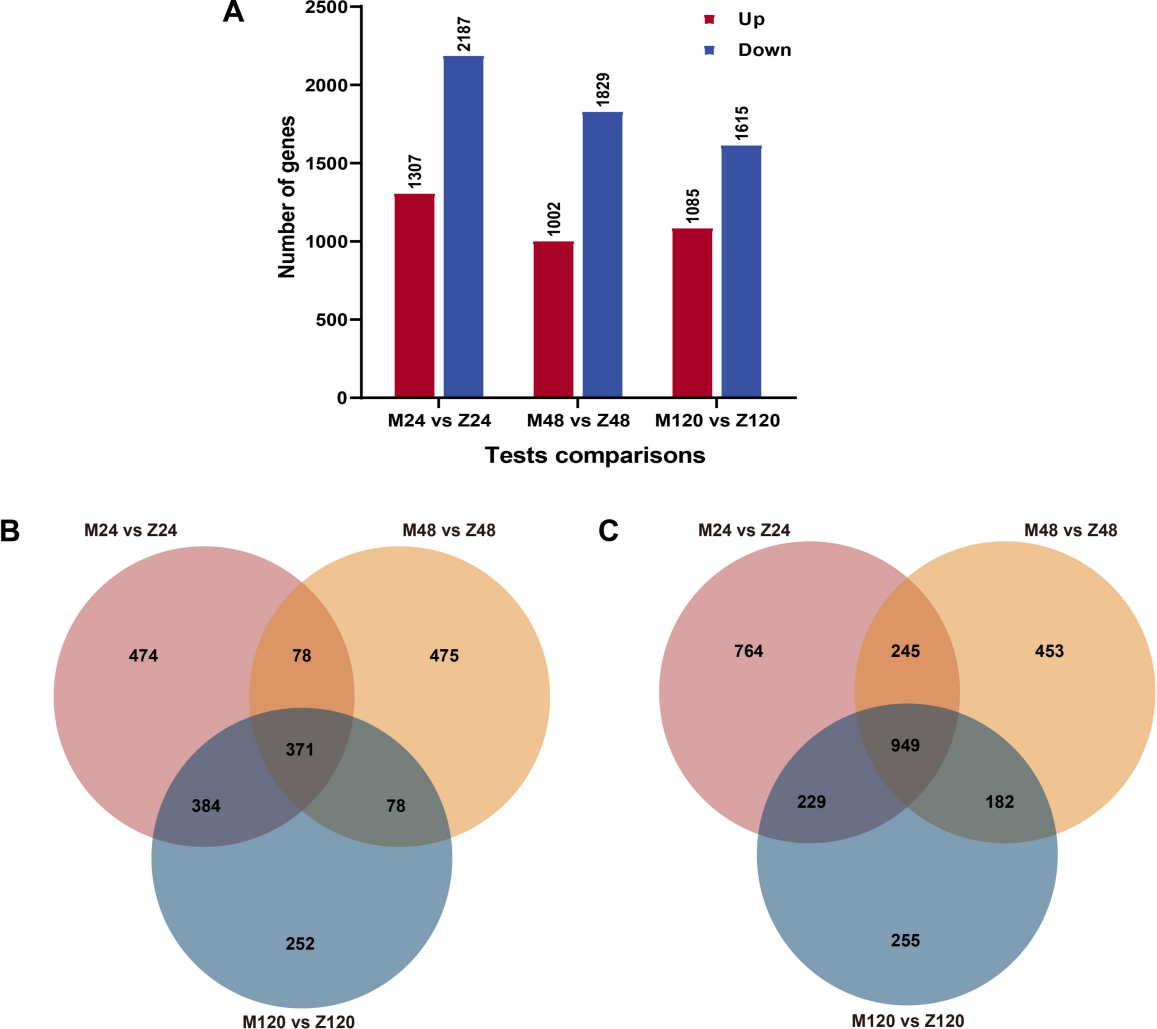
Statistical chart and Venn diagram of the DEGs at the three time points. (A) Statistical chart of the DEGs. DEGs were identified by filtering the twofold upregulated and downregulated genes with a Q-value ≤ 0.001. The rectangles represent the number of upregulated (red) and downregulated (blue) genes. (B) Venn diagram of the upregulated DEGs. DEGs were identified by filtering the upregulated genes with a Q-value ≤ 0.001. These genes were obtained from the M24 vs Z24, M48 vs Z48, and M120 vs Z120 comparisons. (C) Venn diagram of the downregulated DEGs. DEGs were identified by filtering the downregulated genes with a Q-value ≤ 0.001. These genes were obtained from the M24 vs Z24, M48 vs Z48, and M120 vs Z120 comparisons.

To analyze the differential genes present in highly susceptible and highly resistant wheat varieties at different times of inoculation, we performed transcriptome sequencing. Based on the results of the transcriptome data, we set | log2foldchange (FC) | >1 and Q-value ≤ 0.001 as the criteria for screening differential genes. The results revealed that for the 24 h inoculation group, there were 3,494 differential genes (1,307 upregulated genes and 2,187 downregulated genes); for the 48 h inoculation group, there were 2,831 differential genes (1,002 upregulated genes and 1,829 downregulated genes); and for the 120 h inoculation group, there were 2,700 differential genes (1,085 upregulated genes and 1,615 downregulated genes) (Fig. 1A). The subgroup “M24 vs Z24” had the highest number of up- and downregulated genes as well as the total number of differential genes, followed by the subgroup “M48 vs Z48.” To identify potential susceptibility-related genes based on MX169’s high susceptibility, this study focused on differentially expressed genes that were upregulated in MX169. The upregulated differential genes in the 24, 48, and 120 hpi groups were compared, and 371 genes that were upregulated were found to be common across all three groups (Fig. 1B). On the other hand, we also compared the downregulated genes at different inoculation times (Fig. 1C).

### Functional classification of DEGs

**Fig 2:**
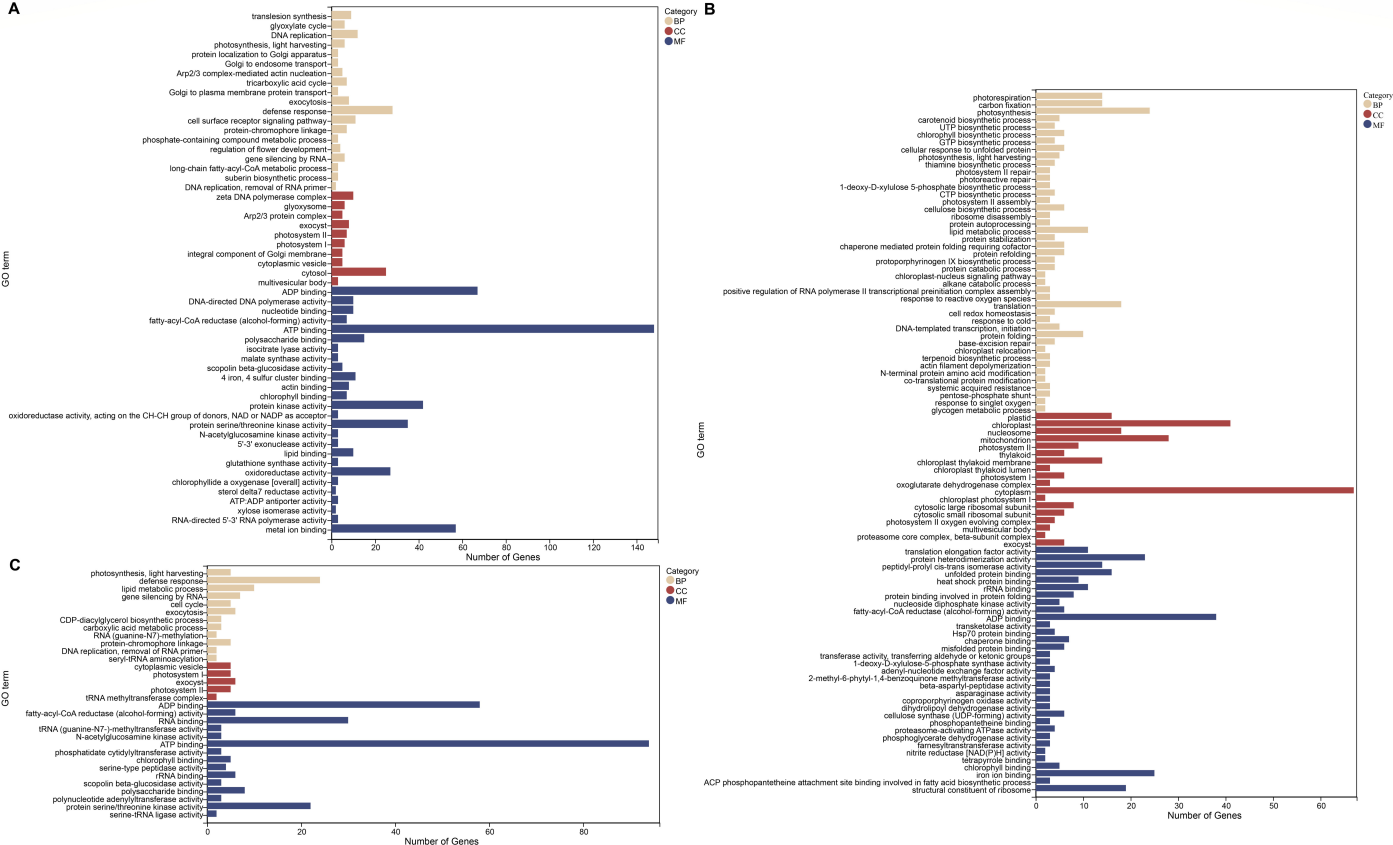
GO annotation of the upregulated DEGs. GO classification of the upregulated DEGs M24 vs Z24 (A), M48 vs Z48 (B), and M120 vs Z120 (C). GO terms were identified by filtering with a *P* value ≤ 0.05.

In order to identify the possible susceptibility genes associated with stripe rust in susceptible varieties, we performed GO analysis on the upregulated DEGs generated at different inoculation time points obtained previously. The DEGs were classified into three categories: molecular functions, biological processes, and cellular components. The number of different GO terms enriched at each time point is shown in Fig. 2. The most enriched GO items in each group were found in the molecular function category, and the most enriched GO items were found in the 48 h post-inoculation group.

When we compared the GO terms enriched to three inoculation time points, seven GO terms were identified to be common. They were photosynthesis, light harvesting (GO:0009765) in the biological process; photosystem I (GO:0009522), photosystem II (GO:0009523), and exocyst (GO:0000145) in the cellular component; ADP binding (GO:0043531), fatty-acyl-CoA reductase (alcohol-forming) activity (GO:0000145), and chlorophyll binding (GO:0016168) in the molecular function.

### KEGG pathway analysis of the DEGs

To further understand the active metabolic pathways involved in the upregulated DEGs identified in the comparison of MX169-vs-Zhong4 at different time points of inoculation, we performed KEGG enrichment analysis, and the results are shown in Table 1. A total of 12 pathways were found to be significantly enriched. At 24, 48, and 120 hpi, upregulated DEGs were found to be co-enriched in the photosynthesis-antenna proteins (ko00196) pathway and significantly enriched at 24 and 120 hpi. In contrast, upregulated genes were enriched in glyoxylate and dicarboxylate metabolism (ko00630) and carbon metabolism (ko01200) pathways only at 24 and 48 hpi, and significantly enriched at 48 hpi. The upregulated genes were enriched in endocytosis (ko04144) at 48 and 120 hpi. Pyruvate metabolism (ko00620) and caffeine metabolism (ko00232) were only present at 24 hpi; upregulated genes were significantly enriched at 48 hpi in carbon fixation in photosynthetic organisms (ko00710), photosynthesis (ko00195), carotenoid biosynthesis (ko00906), biosynthesis of cofactors (ko01240), biosynthesis of secondary metabolites (ko01110), flavonoid biosynthesis (ko00941), thiamine metabolism (ko00730), glycine, serine, and threonine metabolism (ko00260), ribosome (ko03010), and metabolic pathways (ko01100). These activated pathways may play a major role in the differential response of susceptible varieties to *Pst* infection. Among different time points during the interaction between the resistant wheat variety and *Pst*, the 48 hpi time point exhibited a higher abundance of differential gene expression. Furthermore, the significantly enriched pathways at 48 hpi showed greater interconnection with those observed at 24 and 120 hpi.

**TABLE 1:** KEGG pathway enrichment analysis of the upregulated DEGs (M24 vs Z24, M48 vs Z48, and M120 vs Z120)

	Pathway ID	Pathway name	Term candidate gene no.	Total candidate gene no.	Term gene no.	Rich factor	*P*-value	Q-value
24	ko00196	Photosynthesis-antenna proteins	6	112	92	0.0652	2.75E-04	0.0239
	ko00630	Glyoxylate and dicarboxylate metabolism	7	112	240	0.0292	0.0039	0.1714
	ko01200	Carbon metabolism	12	112	804	0.0149	0.0117	0.3380
	ko00620	Pyruvate metabolism	7	112	337	0.0208	0.0190	0.4133
	ko00232	Caffeine metabolism	2	112	7	0.2857	0.0429	0.7456
48	ko00630	Glyoxylate and dicarboxylate metabolism	19	181	240	0.0792	4.21E-11	3.12E-09
	ko00710	Carbon fixation in photosynthetic organisms	16	181	234	0.0684	1.60E-08	5.94E-07
	ko01200	Carbon metabolism	24	181	804	0.0299	6.71E-06	1.66E-04
	ko00195	Photosynthesis	12	181	234	0.0513	2.69E-05	4.98E-04
	ko00906	Carotenoid biosynthesis	6	181	87	0.0690	0.0019	0.0259
	ko01240	Biosynthesis of cofactors	19	181	837	0.0227	0.0021	0.0259
	ko01110	Biosynthesis of secondary metabolites	72	181	5240	0.0137	0.0025	0.0260
	ko00941	Flavonoid biosynthesis	8	181	197	0.0406	0.0040	0.0366
	ko00730	Thiamine metabolism	5	181	68	0.0735	0.0050	0.0409
	ko00260	Glycine, serine, and threonine metabolism	8	181	213	0.0376	0.0060	0.0446
	ko00196	Photosynthesis-antenna proteins	5	181	92	0.0543	0.0142	0.0954
	ko03010	Ribosome	19	181	1041	0.0183	0.0184	0.1137
	ko01100	Metabolic pathways	102	181	8808	0.0116	0.0433	0.2464
	ko04144	Endocytosis	10	181	468	0.0214	0.0488	0.2577
120	ko00196	Photosynthesis-antenna proteins	5	77	92	0.0543	6.45E-04	0.0439
	ko04144	Endocytosis	6	77	468	0.0128	0.0497	1

### Gene set enrichment analysis of the differential genes upon *Pst* inoculation

**Fig 3:**
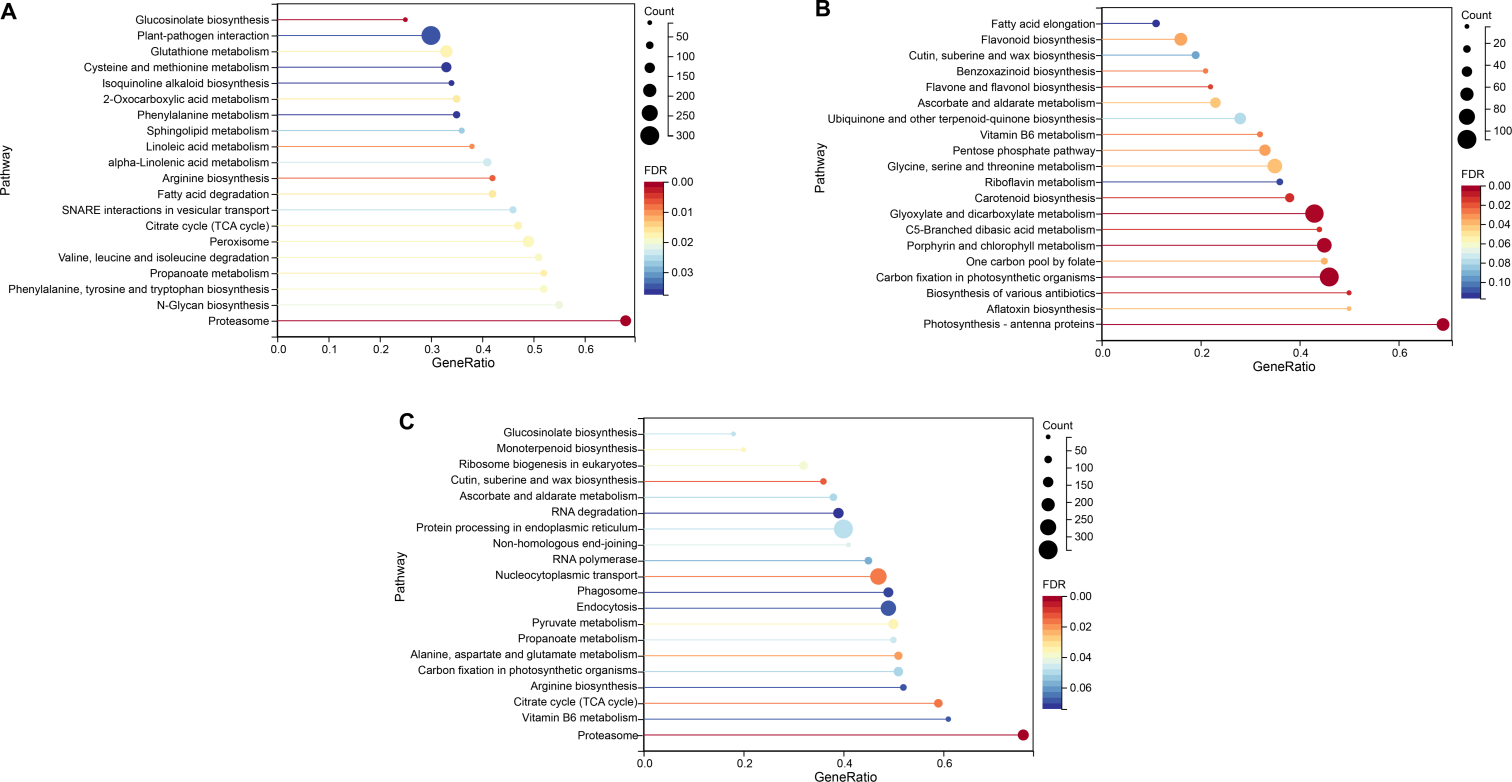
GSEA of the three compared DEGs. GSEA of the DEGs M24 vs Z24 (A), M48 vs Z48 (B), and M120 vs Z120 (C). The set of genes under the pathway at *P* < 0.05, FDR < 0.25 is significant. The top 20 significant enrichment KEGG pathways were obtained from the M24 vs Z24, M48 vs Z48, and M120 vs Z120 comparisons.

**Fig 4:**
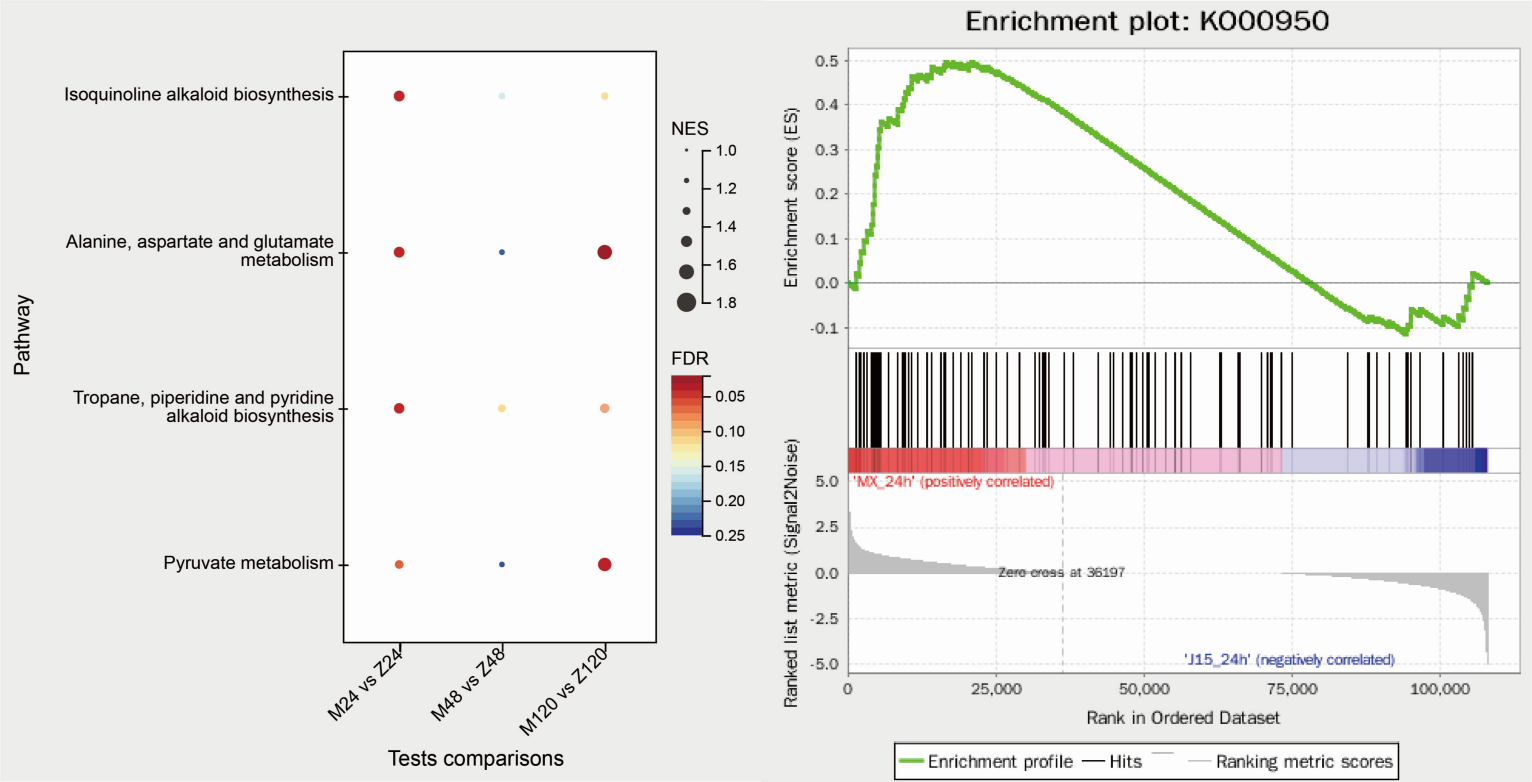
GSEA of the four common pathways in three comparisons. (A) Four common pathways in M24 vs Z24, M48 vs Z48, and M120 vs Z120 comparisons. (B) Enrichment plot of isoquinoline alkaloid biosynthesis (ko00950).

Gene set enrichment analysis can analyze more biologically meaningful gene information and can further help researchers find gene expression-related patterns. Therefore, we analyzed the differential genes at 24, 48, and 120 hpi (*P* < 0.05, FDR < 0.25). We found 43, 26, and 45 significantly enriched KEGG pathways (Table S1), and the first 20 pathways are shown in Fig. 3. We compared them with the pathways in the previous enriched KEGG analysis pathways (*P* < 0.05). At 24 hpi, the shared pathways were caffeine metabolism, carbon metabolism, and pyruvate metabolism; glyoxylate and dicarboxylate metabolism, carbon fixation in photosynthetic organisms, carotenoid biosynthesis, flavonoid biosynthesis, and glycine, serine, and threonine metabolism were shared pathways at 48 hpi; and *the endocytosis pathway occurs simultaneously at 24 and 120 hpi*. A total of four common pathways were identified in the comparison of GSEA at the three time points (Fig. 4). They were isoquinoline alkaloid biosynthesis (ko00950), alanine, aspartate, and glutamate metabolism (ko00250), tropane, piperidine, and pyridine alkaloid biosynthesis (ko00960), and pyruvate metabolism. In the comparison of 24 and 120 hpi, 48 and 120 hpi, 21 and 6 enrichment pathways overlapped respectively.

### Identification of transcription factors

**Fig 5:**
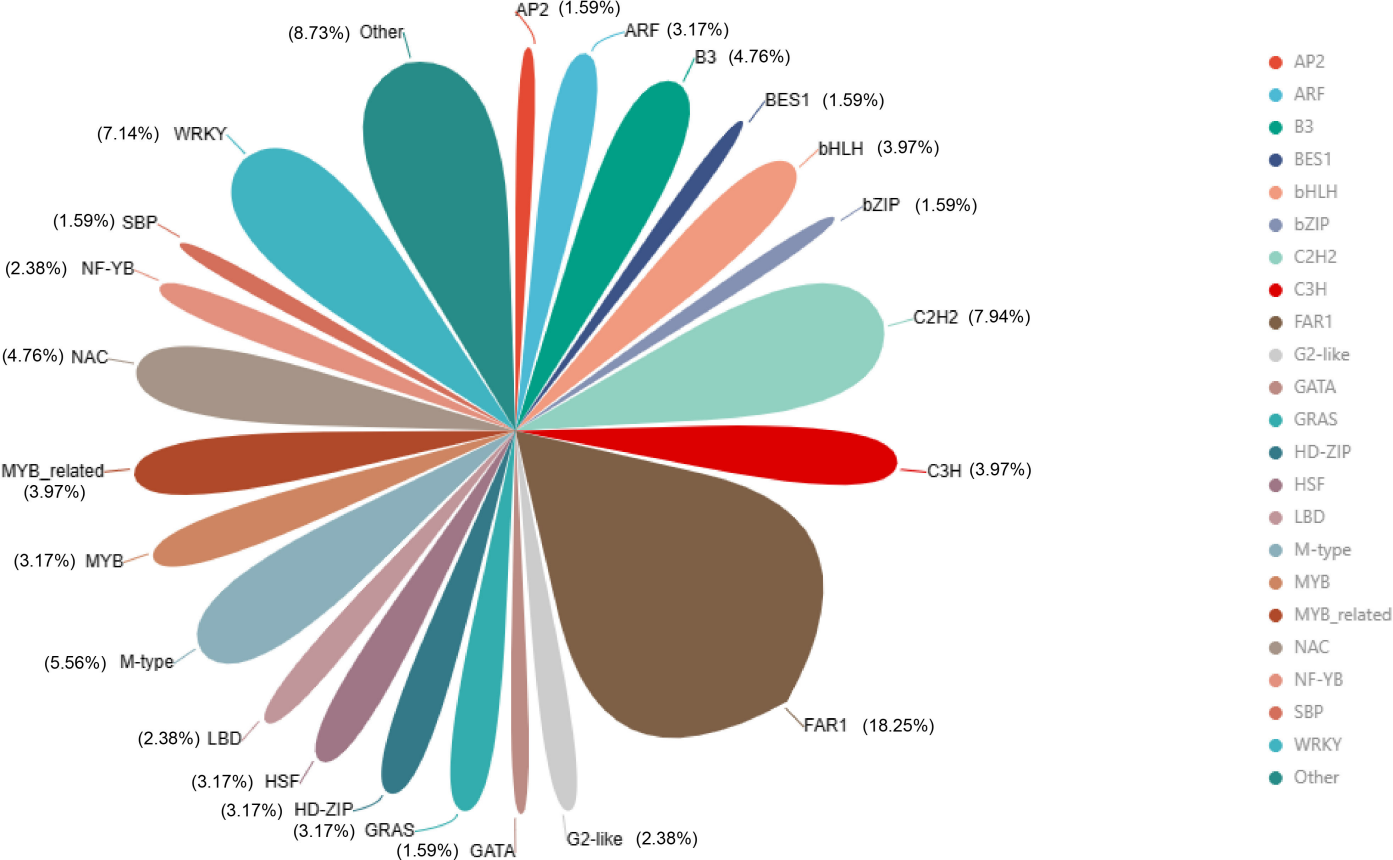
Analysis of upregulated expressed transcription factors shared in all comparisons: 1. AP2 (APETALA2), 2. ARF (auxin response factor), 3. B3, 4. BES1 (BRI1-EMS-suppressor1), 5. bHLH (basic helix-loop-helix), 6. bZIP (basic region/leucine zipper), 7. C2C2 (C2H2 zinc-finger), 8. C3H (p-coumarate 3-hydroxylase), 9. FAR1 (fatty acyl-CoA reductase 1), 10. G2-like (Golden2-like), 11. GATA, 12. GRAS, 13. HD-ZIP (homeodomain-leu zipper), 14. HSF (heat shock transcription factor), 15. LBD (lateral organ boundaries domain), 16. M-type, 17. MYB (V-myb avian myeloblastosis viral oncogene homolog), 18. MYB related, 19. NAC (no apical meristem, ATAF1/2, cup-shaped cotyledon), 20. NF-YB (nuclear transcription factor Y subunit beta), 21. SBP (Squamosa promoter binding protein), 22. WRKY.

Transcription factors (TFs) have important regulatory roles in plant growth and development processes, and plant defense mechanisms. The study of transcription factors can provide an important theoretical basis for basic research and production applications. In this study, 371 shared upregulated genes obtained from inoculation with 24, 48, and 120 h were compared (Fig. 5). Fatty acyl-CoA reductase 1 (FAR1) transcription factors were the most common, and they were encoded by 23 DEGs, accounting for 18.25% of the total. They were followed by C2H2 (C2H2 zinc-finger) transcription factors (10 DEGs, 7.94%), WRKY transcription factors (9 DEGs, 7.14%), M-type transcription factors (7 DEGs, 5.56%), and NAC (no apical meristem, ATAF1/2, cup-shaped cotyledon) transcription factors (6 DEGs, 4.76%).

### Screening and qRT-PCR analysis of candidate disease-susceptibility genes in MX169

Combining the highly significant differential gene (Q ≤ 0.001) and KEGG analysis, we summarized 11 candidate genes associated with susceptibility to stripe rust among upregulated differential genes that co-occurred at three inoculation time points (Table 2). They can be roughly classified into several categories related to plant cell metabolism like carbon metabolism, photosynthesis, and secondary metabolite biosynthesis; plant genetic information processing such as vesicle transport and protein hydrolysis; MAPK pathways in response to pathogen stress and so on.

**TABLE 2:** Candidate genes implicated in the susceptible response to *Pst*

Gene ID	Pathway ID	KEGG pathway	Gene description	Reference
TraesCS3B02G410300	ko04130	SNARE interactions in vesicular transport	VAMP-like protein, putative, expressed	(36–38)
TraesCS2B02G492100	ko00562	Inositol phosphate metabolism	CDP-diacylglycerol-inositol 3-phosphatidyltransferase	(39–41)
	ko01100	Metabolic pathways		
	ko04070	Phosphatidylinositol signaling system		
	ko00564	Glycerophospholipid metabolism		
TraesCS5B02G017800	ko04120	Ubiquitin-mediated proteolysis	Anaphase-promoting complex subunit 5	(42–46)
TraesCS6B02G192400	ko04016	MAPK signaling pathway-plant	Heavy metal P1B-type ATPase, Cu-transporting ATPase, control of Cu accumulation in rice grain	(47, 48)
TraesCS3D02G530700	ko03022	Basal transcription factors	Transcription initiation factor IIA subunit 2	(49–51)
TraesCS4A02G359000	ko03450	Non-homologous end-joining	Flap endonuclease 1	(52–54)
	ko03410	Base excision repair		
	ko03030	DNA replication		
TraesCS6B02G084400	ko00710	Carbon fixation in photosynthetic organisms	EMB3119	(55, 56)
	ko01200	Carbon metabolism		
	ko01230	Biosynthesis of amino acids		
	ko00030	Pentose phosphate pathway		
	ko01110	Biosynthesis of secondary metabolites		
	ko01100	Metabolic pathways		
TraesCS2B02G490800	ko03060	Protein export	Signal recognition particle 14 kDa protein	(57)
TraesCS7B02G482000	ko00906	Carotenoid biosynthesis	Phytoene synthase 7B	(58–60)
	ko01110	Biosynthesis of secondary metabolites		
	ko01100	Metabolic pathways		
TraesCSU02G167700	ko00196	Photosynthesis-antenna proteins	Chlorophyll a-b binding protein, chloroplastic	(61, 62)
	ko01100	Metabolic pathways		
TraesCS3D02G012100	ko04146	Peroxisome	Fatty-acyl-CoA reductase	(63–65)
	ko00073	Cutin, suberine, and wax biosynthesis		

In order to verify the reliability of the transcriptome sequencing results, we selected five candidate disease-susceptibility genes related to wheat stripe rust and analyzed their expression levels at the three time points by qRT-PCR. The results of these genes were consistent with the transcriptome data, although the log2 (fold change) had more or less difference (Table S3; Fig. S1). It indicated that the RNA-seq results of the present study were reliable for all kinds of analysis.

## DISCUSSION AND CONCLUSION

As a widely prevalent disease, wheat stripe rust has a huge impact on the yield and quality of wheat around the world. Currently, it is widely accepted that the most effective method of control is to cultivate and breed durable disease-resistant wheat varieties. The traditional breeding methods using resistance (*R*) genes have limitations due to the pathogen’s continuous mutation. Therefore, disrupting susceptibility (*S*) genes has been proposed as a new approach for breeding durable disease resistance in hosts (66). In recent years, *S* genes have been increasingly widely reported. However, few reports have explored *S* genes from the perspective of wheat, which is highly susceptible to stripe rust (67, 68).

With the increasing sensitivity of sequencing technologies, transcriptome data have been widely used in exploring plant–pathogen interactions (69). It is important to detect possible *S* genes and their transcriptional activity differences in wheat using transcriptome sequencing technology to understand the mechanisms involved in the interaction between highly susceptible wheat and fungi. Therefore, we compared the expression profiles of highly susceptible and highly resistant wheat varieties in response to *Pst*. We identified some potential *S* genes and key metabolic pathways that were significantly expressed in highly susceptible varieties.

In this investigation, we conducted a comparative transcriptome analysis of the highly susceptible wheat cultivar MX169 and the highly resistant wheat cultivar Zhong4 at 24, 48, and 120 hpi. The results revealed a greater number of downregulated differential genes than upregulated genes at different time points, which may be attributed to varietal differences or the enhanced response of resistant cultivars to resist pathogen invasion. The maximum number of differentially expressed genes was observed at 24 hpi, consistent with previous studies on other plant–pathogen interactions. Tang et al. and Van de Mortel et al. have reported a peak in the number of differentially expressed genes at 24 hpi in response to *Pst* infection in resistant varieties and in the soybean–Asian soybean rust interactions mediated by *Rpp2*, respectively (70, 71).

GO analysis of the differentially upregulated genes showed similar results for all three time points, with the genes primarily involved in binding, kinase activity, and catalytic activity in molecular functional ontology, the cellular component in cellular fractions, chloroplasts, and other organelles, and were mainly involved in biological processes such as cellular processes, biosynthesis, and metabolism. Notably, some upregulated differentially expressed genes were enriched in the cell surface receptor signaling pathway (GO:0007166), suberin biosynthesis process (GO:0010345), and defense response (GO:0006952); some of the small molecule compounds and secondary metabolites synthesis process (GO:0009228, GO:0016117) and response to reactive oxygen species (ROS) (GO:0000302) were enriched at 48 hpi, and at 120 hpi, defense response (GO:0006952) was also enriched. Coram et al. analyzed the *Yr5* and *yr5*-mediated expression profiles of non-affinity and affinity wheat*-Pst* interactions and found that basal resistance was similarly expressed in affinity interactions through protein kinases, oxidative bursts, and phenylpropanoid synthesis (such as lignin) (72). This was similar to our results from the GO analysis of upregulated differentially expressed genes.

Following the analysis of differentially upregulated genes using the KEGG method, we found significant enrichment of partial photosynthetic energy metabolism (ko00196, ko00195, ko00710), glyoxalate and dicarboxylate metabolism (ko00630), and thiamine metabolism (ko00730) at different time points during *Pst* infection. Previous studies have revealed that some of these pathways may be involved in the response of wheat to pathogen-induced stress. In particular, photosynthesis is not only an energy metabolism process but also plays a role in regulating plant defense responses induced by pathogen infection (73). Carbon metabolism, glyoxylate and dicarboxylate metabolism, and carbon fixation in photosynthetic organisms have been mentioned to play an important role in the response of the hexaploid wheat line N9134 to *Pst* stress (74). The enrichment of the small molecule compound thiamine metabolism pathway during the *Pst* infection stage has also been confirmed in the study by Hao et al. (75). Furthermore, we used the GSEA method to annotate the upregulated differential genes and found several significant enrichment pathways at all three time points, including pyruvate metabolism (ko00620), alanine, aspartate, and glutamate metabolism (ko00250), isoquinoline alkaloid biosynthesis (ko00950), and tropane, piperidine, and pyridine alkaloid biosynthesis (ko00960). Researchers found that pyruvate metabolism was a highly enriched pathway in wheat responding to leaf rust infection (76, 77). It has been shown that plants will shift carbon flow toward phenylpropane synthesis pathways to cope with environmental stress and produce secondary metabolites such as flavonoids, flavonols, and alkaloids to enhance their resistance to various pathogens (78). In both of these analyses, we found the enrichment of small molecule compounds’ biosynthesis processes such as terpenoids (ko00906), flavonoids (ko00944, ko00941), and alkaloids (ko00950, ko00960). Meanwhile, carbohydrate metabolic pathways such as glycolysis/gluconeogenesis (ko00010) and pyruvate metabolism (ko00620) were similarly enriched at multiple time points. Accordingly, we hypothesized that the susceptible varieties would enhance primary and secondary metabolism to express basal resistance and prevent pathogen infection when infected with *Pst*. In addition, this study also enriched for SNARE interactions in vesicle transport (ko04130), MAPK signaling pathway-plant (ko04016), cutin, suberine, and wax biosynthesis (ko00073), plant–pathogen interactions (ko04626), and phenylalanine metabolism (ko00360), which have been mentioned in related studies as being associated with plant defense responses (68, 79–81).

Transcription factors play a crucial role in regulating transcriptional activity and gene expression. In our study, we detected the highest number of *FAR1* transcription factors, which is a positive regulator of chlorophyll biosynthesis (82). In terms of stress response, researchers found that *FAR1* can enhance antioxidant properties and inhibit cell death by positively regulating inositol biosynthesis (83). *WRKY*, *MYB*, *NAC*, and other transcription factors have been identified as important regulators of host plant response to biotic stresses. In the present study, we found that *WRKY*, *NAC*, *bHLH*, *MYB*, and *bZIP* transcription factors played a significant role in the upregulated genes at three time points during *Pst* infection in MX169 leaves. The *WRKY* transcription factor family is an important class of plant transcription factors that can play both positive and negative regulatory roles in plant defense responses (84). A previous study found that overexpression of *AtWRKY33* not only enhanced Arabidopsis resistance to *Botrytis cinerea* and *Alternaria brassicicola* but also enhanced susceptibility to *Pseudomonas syringae* (85). Researchers have also found that transcription factors such as *bHLH3* and *bHLH13* acted as inhibitors of the JA defense pathway and enhanced the susceptibility of Arabidopsis to gray mold (86). The upregulated expression of differential TFs in the highly susceptible variety MX169 compared to the highly resistant variety in Zhong4 after *Pst* inoculation suggested that the highly susceptible variety may regulate metabolism and defense networks through transcription factors in response to stripe rust infestation.

In this study, we found 11 differentially upregulated expressed genes in response to *Pst* infection in highly susceptible varieties at three time points. These genes played important roles in wheat growth and development, metabolism, and stress response. Among them, the *TraesCS3B02G410300*-encoded YKT61 protein plays a key role in maintaining vacuolar biodynamics, Golgi apparatus morphology, and endocytosis (36). YKT61 is primarily located in the cytoplasm and can interact with BRI1 to promote the dynamic circulation of BRI1 in the plasma membrane. It plays a significant role in brassinosteroid signal transduction, thereby enhancing the plant’s ability to respond to external stress (37, 38). Furthermore, the *TraesCS2B02G492100*-encoded CDP-diacylglycerol-inositol 3-phosphatidyltransferase plays a crucial role in the biosynthesis of phosphatidylinositol (39). The phospholipid metabolic pathway regulated by this enzyme is closely related to plant disease resistance (40, 41). The *TraesCS5B02G017800*-encoded E3 ubiquitin ligase anaphase-promoting complex (APC) subunit 5 is a highly complex molecular machine known to catalyze ubiquitination reactions, directing proteins for degradation by the 26S proteasome (42). Research studies on E3 ubiquitin ligase have revealed that it can directly or indirectly impact on plant disease resistance (43, 44). However, studies on APC mainly focus on plant growth and hormone regulation (45, 46), with limited research on its role in plant response to biological stress. Meanwhile, *TraesCS6B02G192400* is upregulated in the MAPK signaling pathway. It regulates the perception and transduction of ethylene signaling involved in disease resistance by affecting the key protein RAN1 of intracellular copper transfer to ethylene receptors (47, 48). *TraesCS3D02G530700* encodes transcriptional initiation factor IIA subunit 2. As a transcription factor, TFIIA has been found that its γ subunit is an essential element for transcriptional regulation of rice genes (49), and partial inhibition of TFIIAγ expression can also enhance the broad-spectrum resistance of rice to blight and bacterial stripe disease (50). A similar gene function has also been observed in citrus (51). In addition, *TraesCS4A02G359000*-encoded FEN1 protein plays an important role in DNA replication and damage repair (52, 53). During pathogen–plant interactions, a series of defense responses are triggered, such as ROS burst and Ca^2+^ influx (54). This gene may affect downstream defense responses of plant–pathogen interaction by encoding FEN1 protein to regulate intracellular DNA damage repair. *TraesCS6B02G084400* is involved in several basic metabolic pathways that are associated with plant–pathogen interaction in various studies (55, 56). Signal recognition particles (SRP) is the basis of protein co-translation to its appropriate membrane localization and secretion pathway; *TraesCS2B02G490800* encodes SRP14. Currently, studies have found that SRP is also involved in regulating many cellular processes, including gene expression, viral infection, apoptosis, and stress response (57). However, most research studies were focused on animal and human samples, with few studies conducted on plant samples. *TraesCS7B02G482000* is significantly upregulated in the carotenoid biosynthesis pathway, which may impact plant development and stress response by affecting photosynthesis (58, 59) and the synthesis of signal molecules such as ABA and strigolactones (SLs) (60). Several studies have shown that the photosynthesis-antenna protein pathway is associated with plant resistance to biological (fungi, viruses, etc.) and abiotic stresses (61, 62). Therefore, the enrichment of *TraesCSU02G167700* in this pathway implies its potential involvement in the interaction between wheat and stripe rust through its effect on photosynthesis. Finally, previous research has shown that the plant cuticle plays a crucial role in the interaction between plants and microorganisms. It can serve not only as a physical barrier between plants and the environment but also as a source of signal molecules for microorganisms and host plants (63). In this study, *TraesCS3D02G012100* was found to be upregulated in the cutin, suberine, and wax biosynthesis pathway. This pathway is related to the biosynthesis of unsaturated fatty acids and encodes alcohol-forming FAR, which is responsible for the metabolism of long-chain acyl CoA to wax. The transcriptome data of peanut leaves infected by *Didymella arachidicola* and tomato seedling leaves inoculated with *Streptomyces lydicus* showed that the upregulated genes were enriched in the cutin, suberine, and wax biosynthesis pathways similarly (64, 65). In conclusion, it is hypothesized that these genes may be involved in plant–host interactions, either directly or indirectly.

Plant–pathogen interactions involve a complex and continuous process that entails the inheritance, variation, expression, and regulation of genes linked to plant resistance and susceptibility and pathogen causation, as well as the interconnection and influence between them. In this study, we explored the potential *S* genes and their mechanisms by comparing the gene expression characteristics of wheat leaves responding to stripe rust invasion under different conditions of compatibility and incompatibility. The comparison between MX169 without any disease resistance genes and high-resistance varieties Zhong4 can maximize the exploration of all possible *S* genes that may cause wheat susceptibility, including background differences. These findings will guide further research on this important field of study. Although transcriptome analysis is a key approach for identifying functional genes, single omics data may not provide a comprehensive depiction of the entire biological process. Therefore, integrating multiple omics techniques such as genomics, transcriptomics, proteomics, and metabolomics can comprehensively elucidate the rules and mechanisms of wheat–stripe rust interactions, providing valuable insights for identifying susceptibility genes and breeding durable resistance varieties.

In summary, the highly susceptible wheat variety MX169 has great potential to find potential susceptibility genes to improve stripe rust resistance with new approaches. We have preliminarily analyzed the potential mechanisms and candidate genes for responding to stripe rust by comparing MX169 with the highly resistant wheat variety Zhong4. The majority of the differentially expressed genes were observed at only one or two time points, and functional analysis of these genes revealed that the highly susceptible varieties may respond to stripe rust infestation through pathways such as photosynthesis, carbon metabolism, and biosynthesis of secondary metabolites. Our hypothesis is that the expression of DEGs involved in these pathways reflects not only the basic resistance expressed by the high-sensitivity variety when it is invaded by the pathogen but also the negative regulatory effects of some genes in these pathways that inhibit resistance and increase disease susceptibility. We also identified some DEGs encoding transcription factors related to plant responses to biotic stress. Our findings provided valuable insights into the transcriptional response of the highly susceptible variety MX169 to stripe rust and offered a theoretical basis for improving wheat resistance to *Pst* from the perspective of susceptibility genes.

## SUPPLEMENTAL MATERIAL

Figure S1 spectrum.03774-23-s0001.docx Expression patterns between qRT-PCR and RNA-seq for the five genes.

Supplemental tables spectrum.03774-23-s0002.xlsx Tables S1-S3.

ASM does not own the copyrights to Supplemental Material that may be linked to, or accessed through, an article. The authors have granted ASM a non-exclusive, world-wide license to publish the Supplemental Material files. Please contact the corresponding author directly for reuse.
