# Supplementary material for: Comparative transcriptomic insights into molecular mechanisms of the susceptibility wheat variety MX169 response to Puccinia striiformis f. sp. tritici (Pst) infection
Source: Microbiol Spectr. 2024 Jun 25;12(8):e03774-23. doi: 10.1128/spectrum.03774-23 (PMC11302261; doi:10.1128/spectrum.03774-23)
Supplement: Figure S1 — Expression patterns between qRT-PCR and RNA-seq for the five genes. [file spectrum.03774-23-s0001.docx]

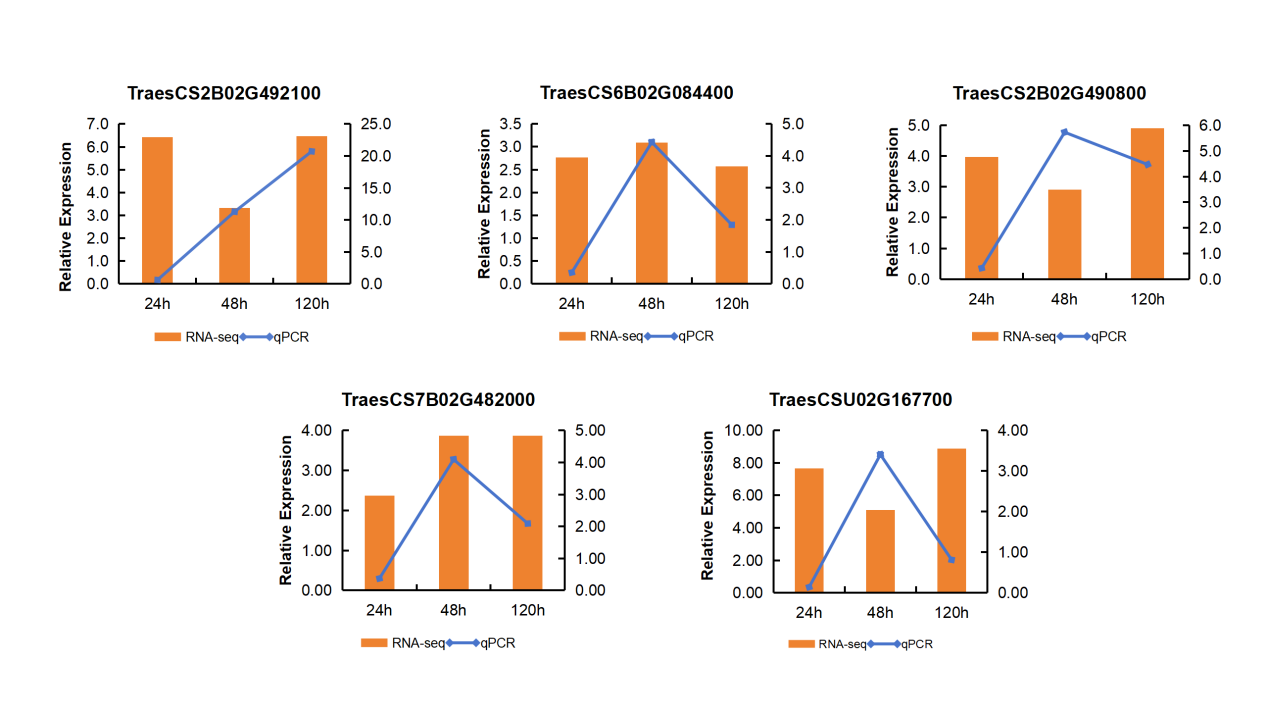


**Fig.S1 Expression patterns between qRT-PCR and RNA-seq for the 5 genes.**Relative gene quantification was calculated by comparative 2^-ΔΔCT^ method. The endogenous β-actin expression level was used as control. The left ordinate axis represents the relative expression level (FPKM) of RNA-Seq, and the right ordinate axis represents the relative expression level of qRT-PCR.
